# Complications and Recurrences after Excision and Reconstruction of Eyelid Tumours

**DOI:** 10.3390/curroncol31040130

**Published:** 2024-03-22

**Authors:** Georgi Balchev

**Affiliations:** Ophthalmology Department, Medical University Pleven, 5800 Pleven, Bulgaria; georgi@balchev.org; Tel.: +359-64-886-657

**Keywords:** oculoplastic surgery, eyelid tumours, basal cell carcinoma, radiotherapy

## Abstract

Introduction: The eyelids are a common site for skin tumours and account for 5–10% of all skin tumours. Treatment is mainly surgical and aims to preserve the anatomical structure of the eyelid, its function and not least its aesthetic appearance. Aim: Presentation of recurrence and complication rates of tumour-related eyelid surgery in a cohort of 450 tumours. Results: Analysis of a cohort of 450 tumours operated on revealed 13 (2.8%) operations with recurrences and 32 (7%) with complications. The statistical significance of recurrences was observed for the involved and uninvolved ciliary margin. At the temporal canthus, 23.1% of recurrences occurred compared to 7.7% at the medial canthus. SGC has the highest recurrence rate. Complications include the following: ectropion, dehiscence, gross cicatrix with normal function, retraction, post-radiation damage, sub-graft haemorrhage and graft rejection. Conclusions: The recurrence rate of eyelid tumours is lower than that of complications. The choice of surgical technique determines the frequency of complications and histological control of the excised tissue, as well as the frequency of recurrences.

## 1. Introduction

The eyelids are a common site for skin tumours and account for 5–10% of all skin tumours [1]. Regarding localisation, different authors show similar percentages, namely the following: 50% of cases are located on the lower eyelid, about 25% on the medial canthus, 15% on the upper eyelid and about 5–8% on the lateral canthus [2,3,4,5]. The localisation and size of the defect determine the surgical approach.

The treatment is mainly surgical and aims to preserve the anatomical structure of the eyelid, its function and not least its aesthetic appearance. The postoperative result should largely fulfil the patient’s cosmetic expectations.

Eyelid tumours are divided into the following three major groups according to their histomorphology on one hand and their invasiveness and tendency to metastasise on the other: benign, malignant and inflammatory tumours (so-called inflammations). A basic classification of tumours based on their histomorphology is proposed by the International Histological Classification of Tumours and published by the WHO [6,7,8]. This classification distinguishes the following main histological groups: 1. epidermal tumours, 2. glandular tumours, 3. stromal tumours and 4. inflammatory lesions resembling neoplasms. Each group is divided into subgroups that cover both the respective histomorphological substructure and the distribution for malignancy.

The most common of all eyelid tumours are those in the first group, epidermal. Of these, 85% belong to one of three groups: benign proliferations, basal cell carcinoma and melanotic lesions. The remaining 10–15% are all other eyelid tumours [4,8,9]. Basal cell carcinoma (BCC) is one of the most common tumours and its incidence is increasing by 10% annually worldwide [3,4]. Of all BCC cases, 80% occur in the head and neck area, and of these, 20% are found in the eyelid area [1,10,11].

Various studies have shown that BCC is the most common eyelid tumour, accounting for 86–91% of cases in Caucasians [12,13]. However, in large Asian studies, the incidence of BCC is lower than that of sebaceous gland carcinoma (SGC), which accounts for 32% of all skin tumours in China and India [14,15] and 67–77% throughout Asia [16,17,18].

### 1.1. Diagnostic Biopsy

A diagnostic biopsy is used to make an accurate diagnosis before the actual surgical procedure. In the case of small lesions that allow a simultaneous excision of 2–3 mm in healthy tissue, excision and biopsy are performed in one step. If the expected defect requires reconstruction or skin grafting, it is possible to perform this in two stages with histological control of the edges of the surgical wound. Excision is carried out until a histologically clear surgical margin is reached. Shave, punch, incision, impression, aspiration and excision techniques are used for the diagnostic biopsy. It is important to note that a diagnostic incisional biopsy should be avoided if malignancy is suspected in order to reduce surgical trauma. An excisional biopsy is usually preferred.

### 1.2. Tumour Excision

Excision of the tumour mass aims to remove the visible tumour completely, together with a small amount of healthy tissue. The aim of “intact” excision is to achieve a surgical defect with tumour-free margins. Different guidelines recommend different sizes of excision in “healthy” tissue, but the average is around 3 mm for carcinomas and around 8 mm for melanomas. Direct tumour excision is the most commonly used technique for eyelid tumours. This creates a surgical defect of varying size that must be properly reconstructed to preserve the function and cosmetics of the eyelids. This is often a challenge and requires the use of various surgical techniques, which are described below.

### 1.3. Aim

The aim of this study was to present the recurrence and complication rates of tumour-related eyelid surgery in a cohort of 450 tumours.

## 2. Materials and Methods

This study is a retrospective follow-up of 450 eyelid tumours over a period of 10 years (2010–2019) that were operated on at the Pleven Eye Clinic. Of all tumours operated on, only those in which a histological examination revealed a benign or malignant tumour were included. All inflammations, such as chalazion, etc., were removed from the sample.

Marking the tumour defect in healthy skin is an important stage in the preoperative evaluation. If the clinical judgment is in the direction of a malignant tumour, the marking is wider; “in healthy” skin it is (3–4 mm) for BCC and SCC, and for MM it is 8 mm or greater. For topographical–anatomical orientation, the histological material should be marked, which is done using different coloured surgical sutures. This provides information on where there is incomplete excision and re-resection is needed.

During surgery, we can only assume that we cut into “a healthy skin”. In case of doubt, the wound is not closed, but left open for a week after thorough coagulation and antibiotic ointment. After the histology comes out, we revise the wound. Then, we are aware of whether the excision lines are tumour-free or not, wherein we need to re-excise according to the marking. MOHS surgery is the best option, but not applicable in our clinic.

Kolmogorov–Smirnov and Shapiro–Wilk tests showed normal data distribution. With analysis of variance (ANOVA/ANCOVA), it was determined whether certain factor variables of the categorical type cause systematic variation in a given variable of the quantitative type. A multivariate analysis was performed for two or more independent variables of the categorical type.

Correlation analysis shows the degree of relationship between the studied indicators. The age and size of the tumour were not categorical types, so ANOVA/ANCOVA analysis was not applicable. Bivariate correlation analysis was performed, and Pearson’s coefficient (r) was calculated. All of the statistical analyses were performed with SPSS, version 26 (IBM Corp, Armonk, NY, USA).

## 3. Results

### 3.1. Recurrences

When analysing a cohort of 450 operated tumours, 13 (2.8%) operations with recurrences and 437 (97.2%) without recurrences were found. The following statistical associations were identified with respect to recurrences.

**Figure 1:**
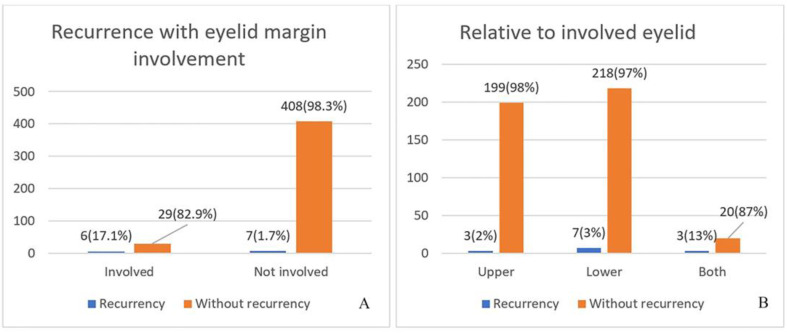
Relapse in involved ciliary margin (*p* < 0.001) (**A**) and with an involved eyelid (*p* < 0.007) (**B**).

The statistical significance of the recurrences was observed for the involved and the uninvolved ciliary margin. The number of recurrences was evenly distributed between the two groups, but in percentage terms, recurrences with an involved eyelid margin accounted for 17.1% of all recurrences, compared to 1.7% with a non-involved margin, which is a large and significant difference. Involvement of the lower eyelid again resulted in more recurrences. At the temporal canthus, 23.1% of recurrences were reported, compared to 7.7% at the medial canthus (*p* < 0.001) (Figure 1).

**Figure 2:**
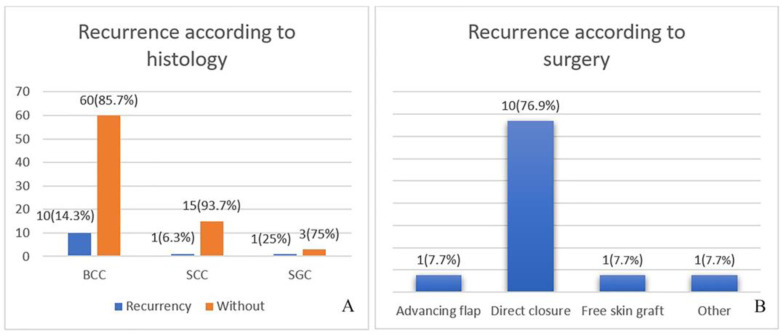
(**A**) Recurrences in relation to histology, (**B**) recurrences in relation to surgical technique (*p* < 0.001).

Regarding histology, the highest percentage of recurrences is found in BCC at 8%, followed by Squamous cell carcinoma (SCC) (1%) and SGC (1%) in equal percentages. Of the histological variants of BCC, the solid variant has the highest percentage of recurrences at 77.4%. The intragroup comparison showed different results. SGC recurred in 25%, BCC in 14.3% and SCC in 6.25%, meaning that SGC—with the highest recurrence rate—was the most common variant (Figure 2A).

The results are interesting in connection to the selected surgical technique. More recurrences were observed with direct closure (76.9%), followed equally by advanced flap and free skin grafting at 7.7% (Figure 2B).

Regarding comorbidity with other malignancies, including non-ophthalmology, we found nine patients with a history of another malignancy, representing 2% of all patients without statistical significance of the overall outcome.

### 3.2. Analysis of Dispersion

The analysis of variance was used to determine whether certain categorical factor variables cause systematic variation in a specific quantitative variable.

**Figure 3:**
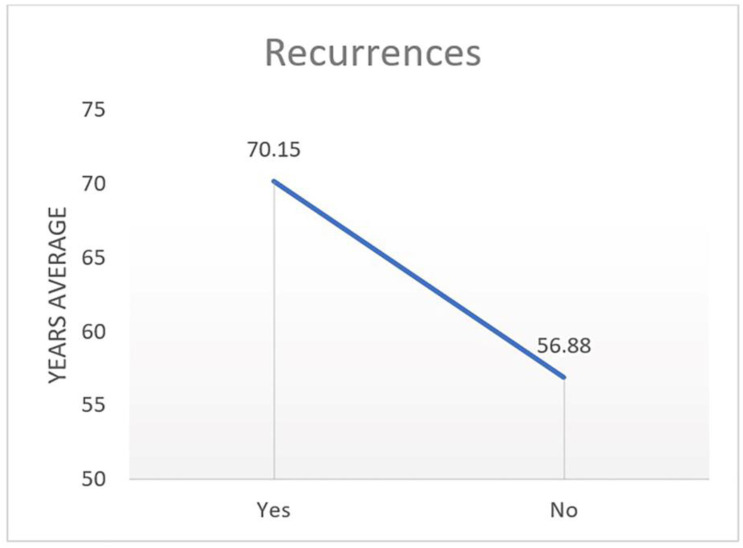
Recurrences/age (*p* < 0.014).

A positive correlation was observed between age and relapse (*p* < 0.014) (*p* < 0.009). Relapses are characterised by a higher average age (Figure 3).

**Figure 4:**
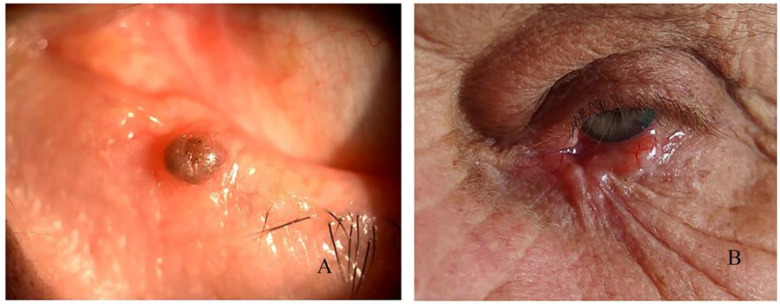
Recurrences: (**A**) nodular, (**B**) causing scarring.

Three recurrences were observed that caused a positional abnormality, which corresponds to 9% of all complications. All of them were diagnosed with basal cell carcinoma (Figure 4).

### 3.3. Complications

The total number of complications for all 450 operations was 32 (7%). They are divided into the following two large groups: direct closure with/without flap at 20 (4%) and plastic at 12 (3%). The complications are associated with impaired eyelid function, with the exception of those classified as “rough cicatrix with normal function”.

**Figure 5:**
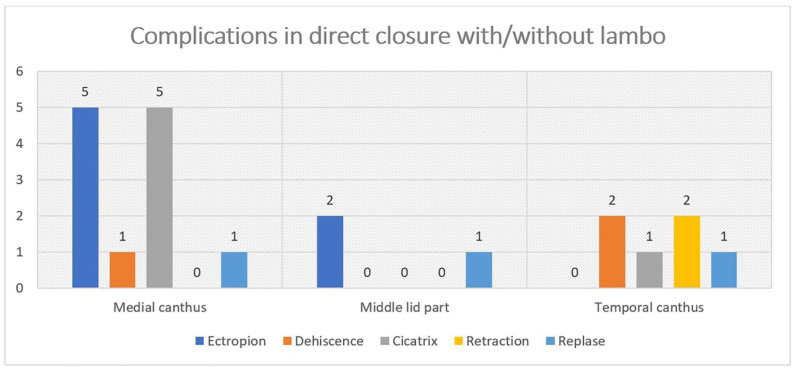
Complications in direct closure or with different types of flaps.

In direct closure with/without flap, the complications observed are the following: **1. Medial canthus**: ectropion, five; dehiscence, one; rough cicatrix with normal function, five and relapse, one; **2. Temporal canthus**: ectropion, one and relapse, one; **3. Middle lid area**: dehiscence, two; rough cicatrix with normal function, one; retraction, two and relapse, one. (Figure 5).

When closing with plastic (including free skin graft), the observed complications are the following: ectropion, five; rough cicatrix with normal function, three; retraction, one and graft rejection, three.

**Figure 6:**
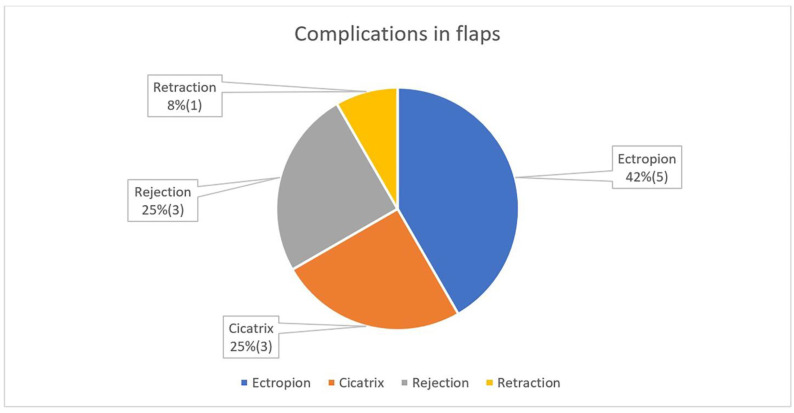
Complications in plastics and other reconstructions.

**Figure 7:**
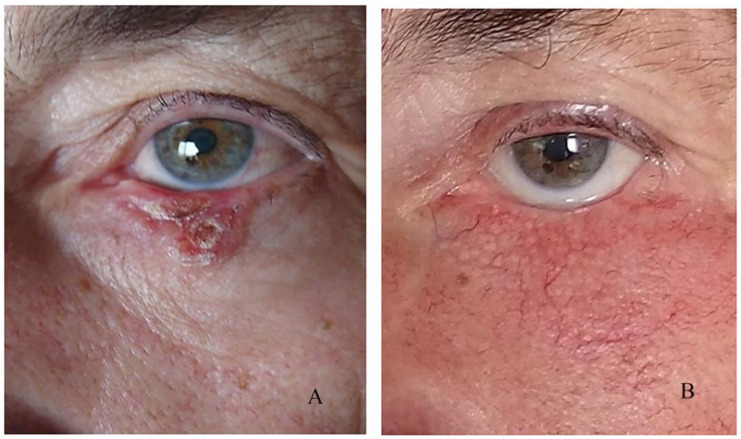
Eyelid retraction due to lack of tissue: (**A**) preoperative, (**B**) postoperative.

Retraction of the lower eyelid was observed in a total of four patients, which corresponds to 12.5% of all complications (Figure 6). This is caused by a lack of tissue to cover the surgical defect, resulting in scarring (Figure 7).

**Figure 8:**
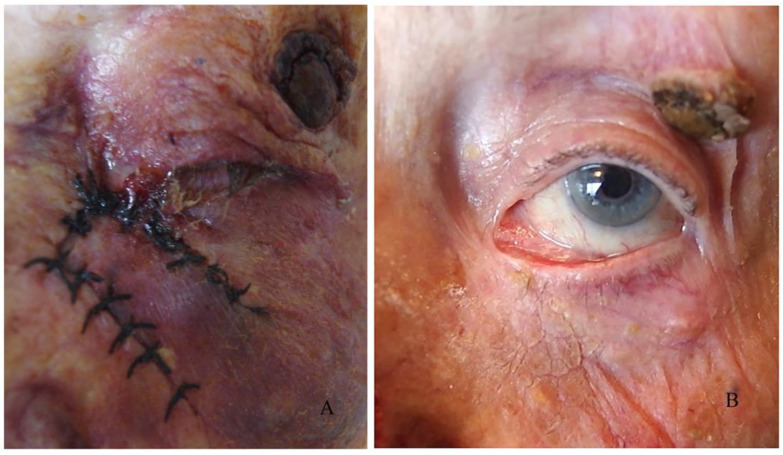
Postoperative ectropion: (**A**) before, (**B**) after.

In cases where only the anterior lamella is missing, the defect is reconstructed with a flap or other skin graft; the graft exerts vertical traction. Ectropion is present (Figure 8) and it was observed in 11 cases, representing 34% of all complications.

**Figure 9:**
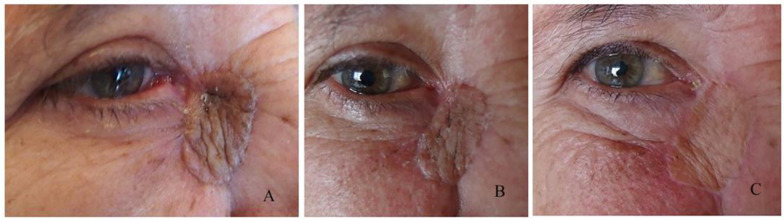
Rough cicatrix in the first months: (**A**) at 1 m, (**B**) at 2 m and (**C**) at 9 m.

Gross cicatrix was observed in nine cases, which was 28% of all complications (Figure 9). It is associated with wound dehiscence, free skin flap and other flaps.

**Figure 10:**
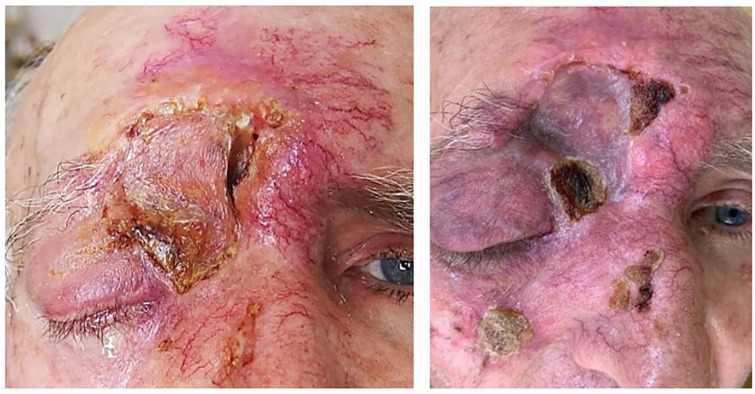
Graft damage after radio therapy.

**Figure 11:**
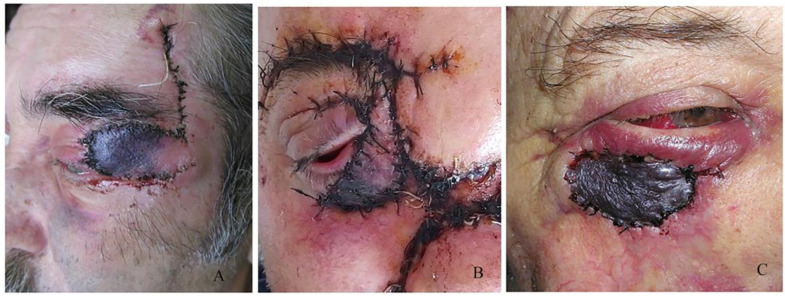
Graft/flap failure after (**A**,**B**) flap necrosis or (**C**) subgraft hemorrhage.

In cases of three patients, there was rejection of the graft, which was 9% of all complications, with one patient having post-radiotherapy graft damage without leading to complete rejection (Figure 10) and another with sub-graft haemorrhage (Figure 11).

All complications were treated surgically, with the goal of reoperation being functional outcome rather than cosmetic.

## 4. Discussion

Depending on their location, eyelid tumours and BCC in particular can quickly invade the surrounding tissue. In the case of tumours in the middle parts of the eyelids, for example, the dermis is affected relatively quickly, whereupon the tumour invades the perichondrium of the tarsus and, if left untreated, exceeds the septum and involves the underlying orbital structures [19].

In medially located tumours of the eyelids, invasion can occur in the direction of the orbital periosteum, onto the medial wall of the orbit and from there into the extraconal orbital fatty tissue. BCC can, for example, invade the eyeball and even reach the base of the skull or the nasal cavities [19].

The localisation and size of eyelid tumours are decisive for the choice of surgical technique. For defects affecting the anterior lamella of the lower or upper eyelid, there is a choice between direct closure, rotational and transposition flaps or musculocutaneous flaps. In cases where the defect also covers the eyelid margin, its size determines the choice of surgical technique. In defects where 30% of the total eyelid tissue is missing, direct closure can still be used. For defects up to 50%, various types of skin or skin-muscle flaps are already used, as well as free skin grafts with or without a tarsal/cartilage graft. For defects over 50%, a combination of skin and skin-muscle flaps is used, as well as transposition of strips from the periosteum, grafts from the adjacent eyelid, the parotid gland region, the hand, the glabella, etc. [20,21,22].

In cases of medial canthal defects, due to anatomical features, a glabellar flap or free skin plastic can be used, and in cases of absence of a posterior lamella, a tarso-conjunctival (allo) graft, cartilage from the ear or nasal septum, hard palate or donor sclera can be used.

### 4.1. Defect Closure

After thorough removal of the tumour lesion in “healthy” tissue, defects of different sizes and locations are formed. Some are easy to close, while others require major surgical preparation. For optimal cosmetic and functional reconstruction, it is necessary to follow some basic rules:

1. Both eyelid lamellae must be surgically restored or reconstructed.
2. They must achieve optimal horizontal stabilization and no vertical traction.
3. The undermining of the skin around the defect should be sufficient so as not to induce tension.
4. Identification of the aponeurosis of the levator muscle and knowledge of the anatomy of the n.facialis are of utmost importance in preserving upper eyelid function.

The main reconstructive techniques for closing the defect are the following:

- spontaneous granulation, “laissez-faire”;
- direct closure;
- cutaneous, cutaneous-muscular, tarso-conjunctival, etc., flaps;
- free transplantation of skin, mucosa, cartilage;
- a combination of techniques.

### 4.2. Early Postoperative Period

For the early postoperative period, we have taken the time from the operation to the seventh day. After surgery, each surgical wound is closed with a sterile dressing for 24 h, after which it is either removed or replaced with a new one. In most cases, the wound is closed with a dressing until day 3, after which it is left open so that it can “breathe”, which contributes to its faster healing. All skin sutures are removed on day 7–8. The removal of the stitches marks the beginning of the late postoperative period.

In the early postoperative period, it is observed mainly for signs of infection. The majority of patients are on local antibiotic therapy. It is rare for patients to be simultaneously on local and systemic therapy. If infection is suspected, the wound is washed daily or treated surgically.

In this period, we monitor the adaptation of the wound edges and the process of healing. In the case of wound dehiscence, which can occur when the sutures have been cut through the wound or if the primary graft/flap adapts poorly to the wound bed, we can refresh the wound edges and replace the sutures.

Another significant point in the early postoperative period is graft or flap survival in patients with a large flap or free skin graft. For the first few days, we observe the colour of the graft/flap. The colour depends on the revascularization and it is pale on the first day. In the following days, it begins to turn pink, and after a few months, it regains its original colour. In cases of skin graft rejection or flap necrosis, instead of pink, the colour of the graft is entirely black. In the flap, the blackening begins from the edges of the wound.

In our reports, we have observed several partial rejections in free skin grafts and one case of subgraft hematoma that resulted in necrosis of the entire graft. In the case of necrosis, if the area is small, we clean the necrotic tissue and let this area heal primarily (in cases with a flap) or replace the entire graft (in cases with a skin graft).

In combined plastics, especially glabellar, in the early postoperative period, the cosmetic result is poor, and surgeons with little experience may worry. In the late postoperative period, when the swelling decreases and the wound heals, we have reported a significant improvement in the cosmetic result.

We did not observe infections in the early postoperative period.

### 4.3. Late Postoperative Period

The late postoperative period begins on the seventh day. Complete healing is individual; in most cases, it is around the sixth month. In tumour surgeries, we focus mainly on the presence of recurrences, not just on the healing period. In the late postoperative period, we monitor the result of the surgical intervention, evaluating it against the anatomical, functional and cosmetic results, as well as the absence of local metastases.

During healing, each surgical wound passes through the phases of reparative inflammation. In the first month, young connective tissue is observed, with a red cicatrix, and in some patients, a more pronounced proliferation with an uneven wide cicatrix. The final phase of wound healing is associated with the transformation of fibrous tissue into mature connective tissue that can contract. The contraction of the wound defect leads to the formation of irregular cicatrices, pulling the surrounding tissues, which in turn leads to a violation of the position of the eyelids. Despite successful surgery, the healing process is individual, and in some patients, it is more pronounced to the extent of a keloid.

Positional anomalies are a significant complication after eyelid tumour surgery. Ectropion, entropion and trichiasis are mainly observed. Mild positional abnormalities may be present, with a positive functional outcome. In this case, additional surgical intervention can be avoided. In cases where we have a good anatomical and functional result but a bad cosmetic one (pigmentation, cicatrix), an additional cosmetic procedure can be started after the sixth month, when we think that the recovery period of the wound is over. This period also varies from patient to patient due to individual wound healing patterns.

Observations for recurrences begin on the third and sixth months after the operation. Particular attention is paid to cases with SGA due to the high recurrence rate of this tumour. In cases with local metastasis in BCC and SCC, re-excision is performed when healthy with histological control of the edges, without waiting for the complete healing of the wound. The moment of detection of local recurrences is an indication of a new operation.

### 4.4. Radiotherapy: “Pros” and “Cons”

Radiation and chemotherapy are significant parts of tumour treatment in general; however, in ophthalmology, the approach differs from that of other clinical disciplines and often does not coincide with them. As a statistic, only a small proportion of all eyelid tumours give distant metastases. Of the most common eyelid tumour, BCC, it locally advanced in less than 1% of cases, and of that 1%, less than 0.2% give distant metastases. SCC is associated with a slightly higher rate of metastases, while SGC and MM metastasize in a higher percentage of cases [3,23,24,25].

Radiotherapy for eyelid tumours is rarely administered, mostly postoperatively, and requires meticulous planning. Histological control of excision is mainly relied upon. After excision, if we find that the tissues in depth or the orbit are affected by the tumour, we could refer the patient for radiotherapy. When the tumour is deeply set and radical excision is not possible, we refer the patient for radiotherapy. In these cases, reconstructive surgery can be divided into the following two stages: excision with subsequent radiotherapy and second-stage reconstruction.

In the setting of a well-reconstructed or transplanted defect, radiotherapy will compromise the healing process. Shrinkage with subsequent necrosis and shedding of the transplanted skin structures, dehiscence of the surgical wound, loosening of the flaps and damage to the surrounding healthy tissues are observed. Tissue healing after radiotherapy is inevitably associated with their contraction. Irradiation—pre- or postoperatively—leads to atrophy of the adjacent skin, and it becomes thin as parchment, which makes it unsuitable for reconstruction. Contraction and atrophy in the eyelid area cause cicatrixes and positional abnormalities, such as ectropion, entropion, etc., which compromise both the physiological and cosmetic outcome; therefore, radiotherapy is unsuitable preoperatively. However, if a decision is made for radiotherapy, direct consultation with a radiologist is appropriate to assess the radiosensitivity of the tumour and the size of the irradiated area.

Depending on the dose and type of radiation used, the effect is destructive or metabolically active [26]. Photon radiation is most often used, with X-rays that penetrate deeply into tissues, including healthy ones, which is why the side effects of this therapy are due. Proton particles also penetrate deeply. Their advantage lies in the fact that they release their charge only in tumour cells and do not damage healthy tissue. It also allows for larger doses of radiation. Proton treatment is more suitable for ophthalmology than X-rays. The disadvantage is the cost of the treatment.

The side effects of radiotherapy are many: the lens and cornea are directly damaged by X-rays despite the precautions taken, which leads to a decrease in vision.

Destructive radiotherapy, chemical or thermal burning as a treatment method for eyelid tumours, are also not a good alternative to surgery due to the lack of histological control of the treated area. It is a prerequisite for missing parts of the irradiated tumour. Histology is of primary importance as it gives information on whether the tumour is radiosensitive and whether the resection lines are clear.

There are many different opinions on the use of radiotherapy in eyelid tumours, and our practice and the world’s practice show a low frequency of preoperative irradiation [26,27,28]. In our study, we have observed a postoperatively irradiated patient who rejected the skin grafts. Due to maceration of the surrounding tissues, new plastic surgery was impossible.

## 5. Conclusions

The recurrence rate of eyelid tumours is low, but the complication rate is higher. The correct choice of surgical technique determines the frequency of complications, while the histological control of the excised tissue determines the frequency of recurrence. In case of a recurrence, new excision begins immediately; however, in the presence of a complication, a wait of 6 months is necessary.

## Data Availability

Data are available on reasonable request from the corresponding author.
